# Supplementary material for: Response to Bovine Viral Diarrhea Virus in Heifers Vaccinated with a Combination of Multivalent Modified Live and Inactivated Viral Vaccines
Source: Viruses. 2023 Mar 8;15(3):703. doi: 10.3390/v15030703 (PMC10054639; doi:10.3390/v15030703)
Supplement: Supplementary file 1 [file viruses-15-00703-s001.zip › viruses-2207717-supplementary.pdf]

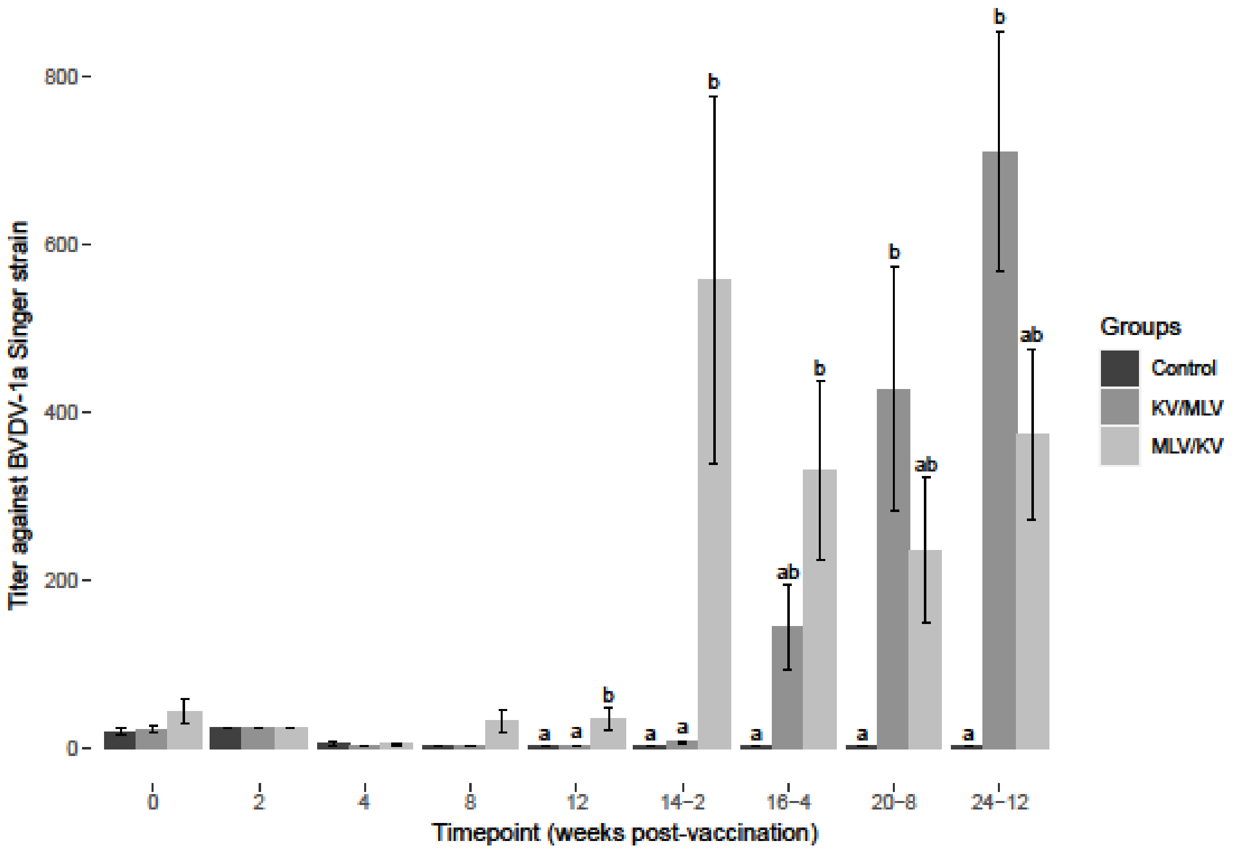

Supplementary Figure S1. Average virus neutralization titers from sera collected at respective timepoints over the course of the study against BVDV-1a (Singer) refence strain. Values at each timepoint, not connected by the same letter are significantly different ($P < 0.05$).

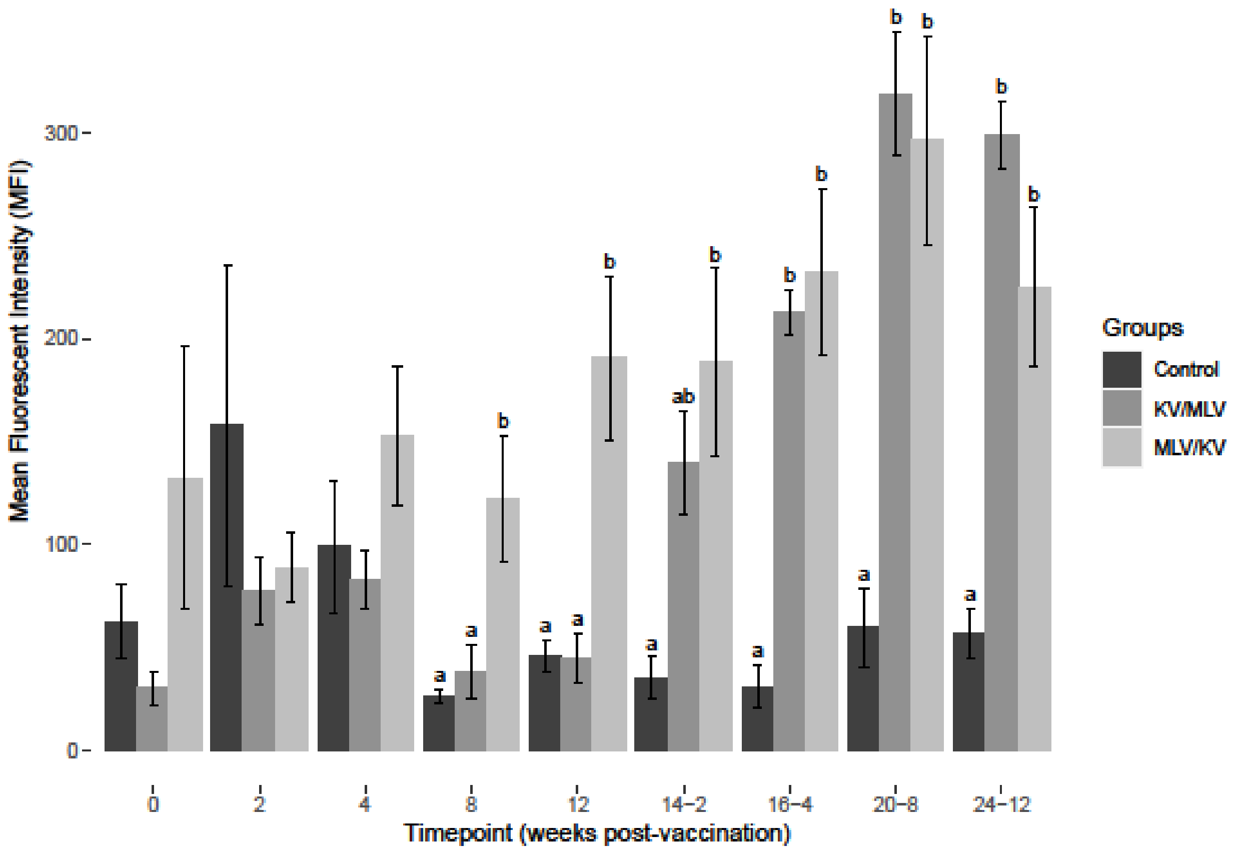

Supplementary Figure S2. Average mean fluorescent intensity (MFI) for CD25+ (IL-2α receptor) PBMC after 24-hour stimulation at each respective timepoint over the course of the study with BVDV-1a (PI-34). Values at each timepoint, not connected by the same letter are significantly different ($P < 0.05$).

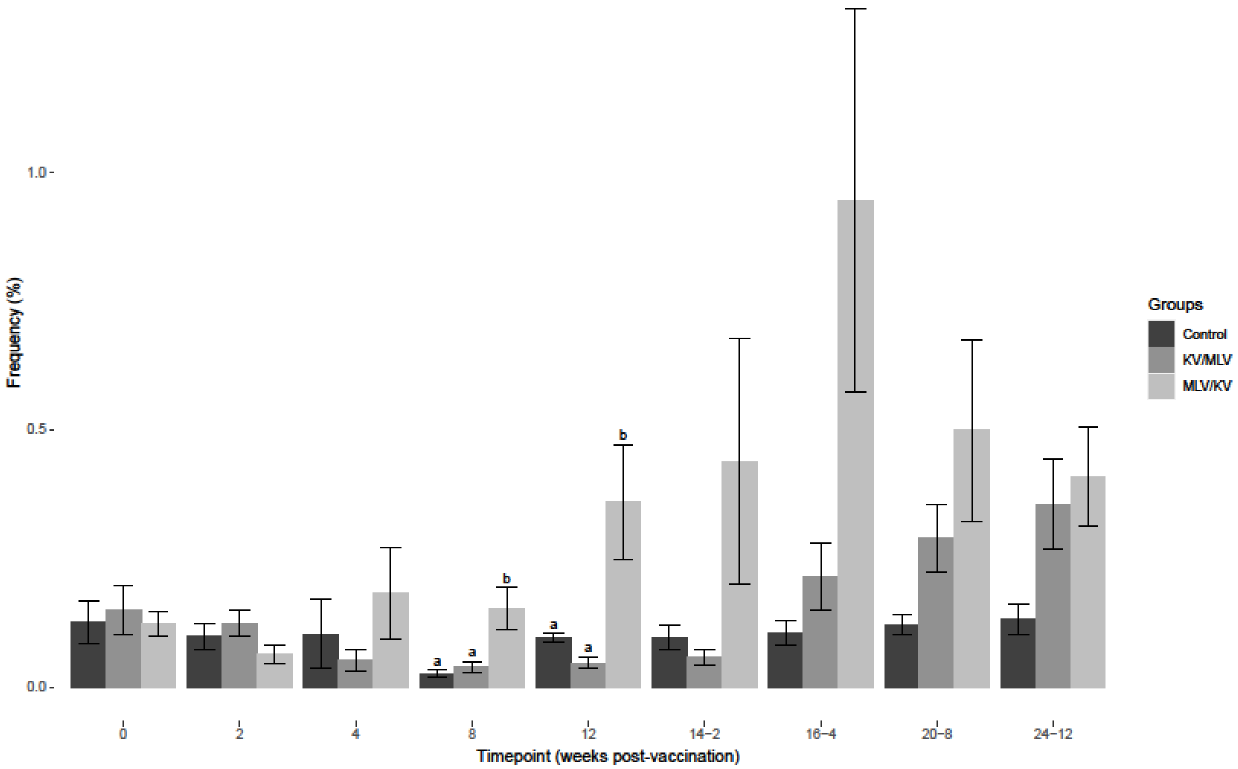

Supplementary Figure S3. Frequency of IFN-γ mRNA-positive $CD4^+$ cells after 24-hour stimulation at each respective timepoint over the course of the study with BVDV-1a (PI-34). Values at each timepoint, not connected by the same letter are significantly different ($P < 0.05$).

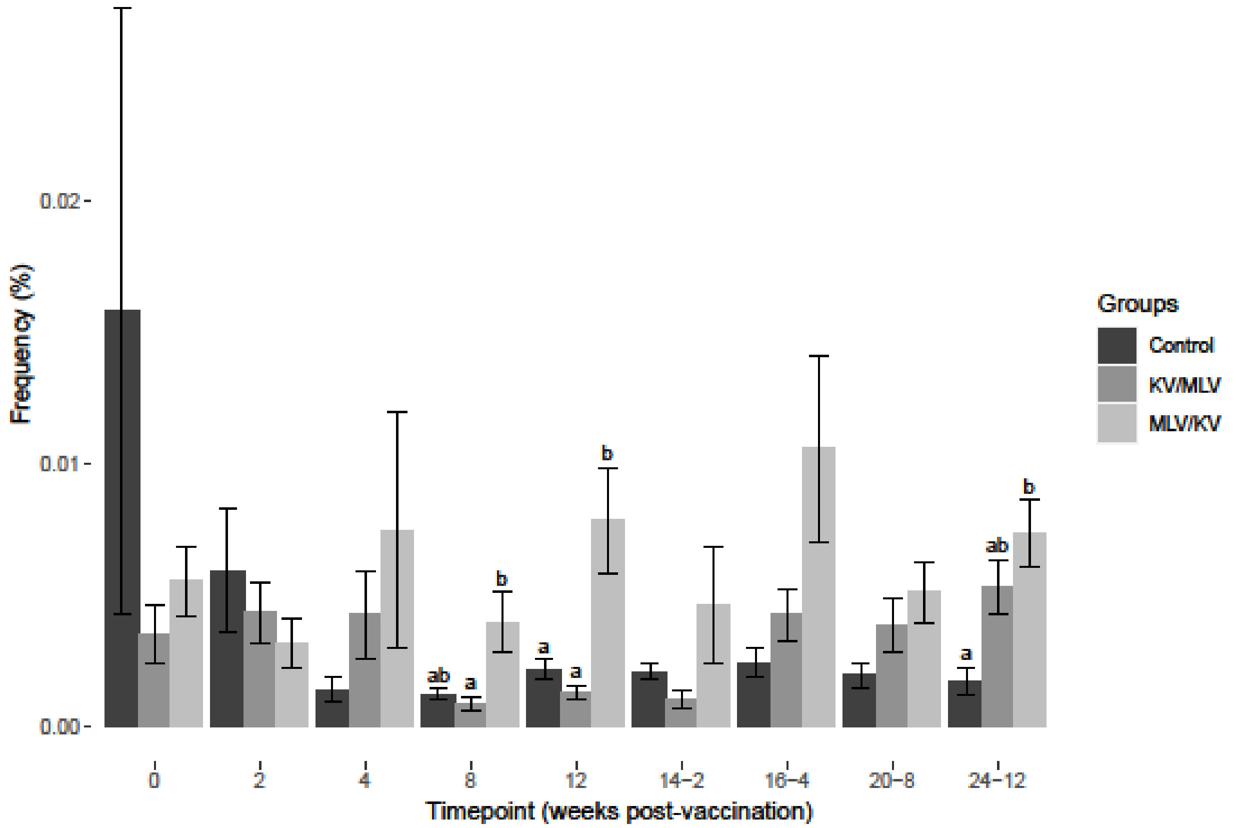

Supplementary Figure S4. Frequency of IFN-γ mRNA-positive CD8+ cells after 24-hour stimulation at each respective timepoint over the course of the study with BVDV-1a (PI-34). Values at each timepoint, not connected by the same letter are significantly different (P < 0.05).

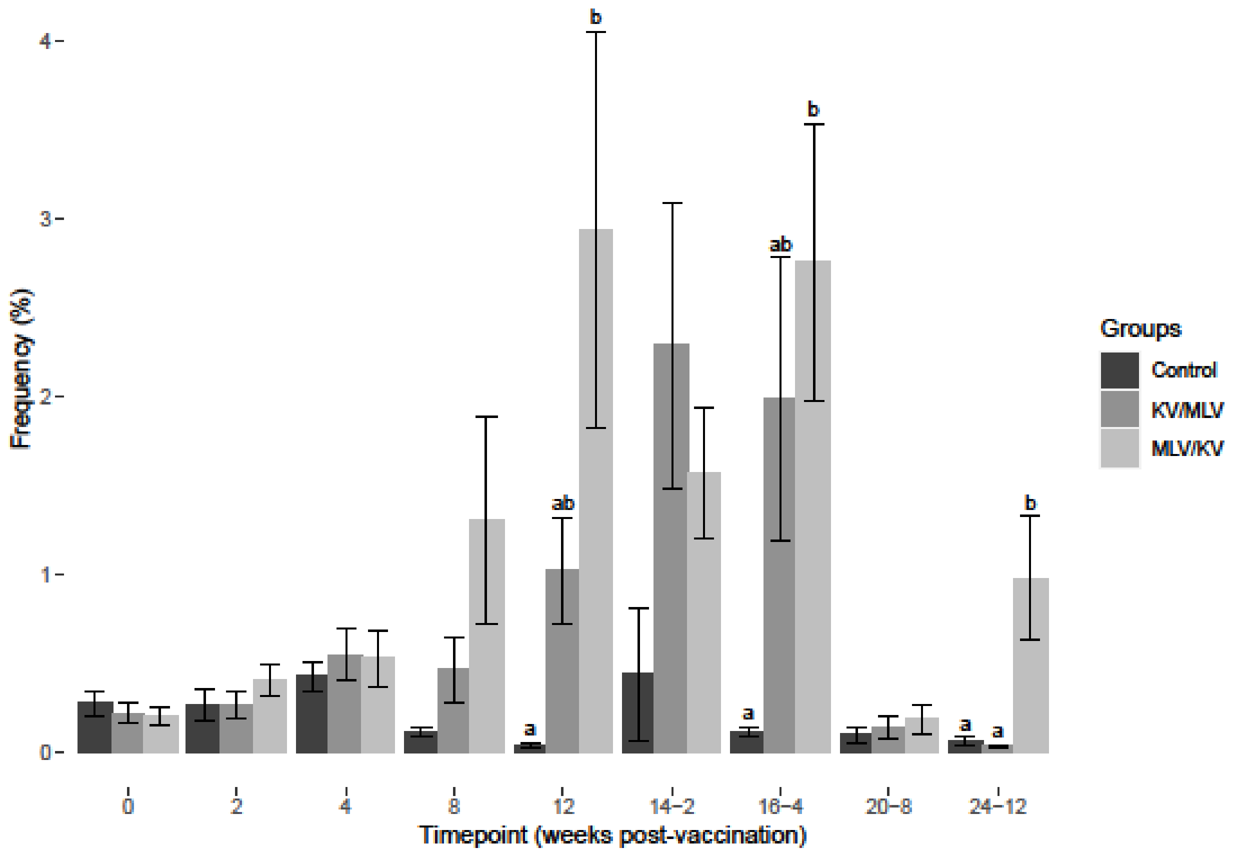

Supplementary Figure S5. Frequency of IFN-γ mRNA-positive CD335[+] cells after 24-hour stimulation at each respective timepoint over the course of the study with BVDV-1a (PI-34). Values at each timepoint, not connected by the same letter are significantly different ($P < 0.05$).

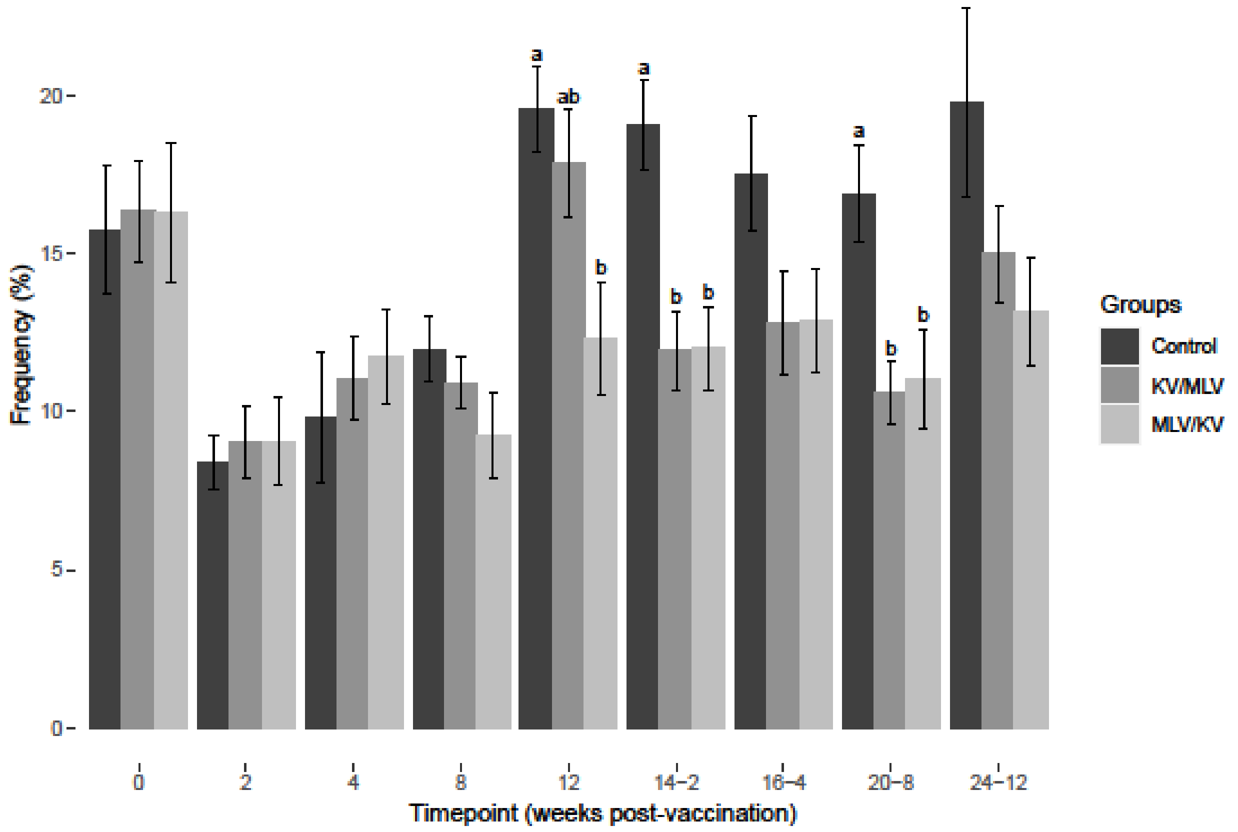

Supplementary Figure S6. Frequency of PBMC positive for the respective BVDV PrimeFlow probe after 24-hour stimulation at each respective timepoint over the course of the study with BVDV-1a (PI-34). Values at each timepoint, not connected by the same letter are significantly different ($P < 0.05$).
